# Treatment optimization of angiotensin converting enzyme inhibitors and associated factors in Ayder Comprehensive Specialized Hospital: a cross-sectional study

**DOI:** 10.1186/s13104-017-2820-5

**Published:** 2018-03-28

**Authors:** Tesfay Mehari Atey, Tsegay Teklay, Solomon Weldegebreal Asgedom, Haftay Berhane Mezgebe, Gebrehiwot Teklay, Molla Kahssay

**Affiliations:** 1 Department of Clinical Pharmacy, School of Pharmacy, College of Health Sciences, Mekelle University, Mekelle, Tigray Ethiopia; 2 School of Pharmacy, College of Health Sciences, Mekelle University, Mekelle, Tigray Ethiopia; 3 School of Public Health, College of Health Sciences, Semera University, Semera, Afar Ethiopia

**Keywords:** Treatment optimization, Angiotensin converting enzyme inhibitors, Heart failure

## Abstract

**Background:**

Angiotensin-converting enzyme inhibitors have morbidity and mortality benefits in heart failure. Failure to optimize treatment using these medications increases hospitalizations, worsens signs and symptoms of heart failure, and reduces the overall treatment outcome. Therefore, the main purpose of this study was to assess the practice of treatment optimization of these medications and associated factors.

**Results:**

A hospital-based cross-sectional study was conducted on 61 ambulatory heart failure patients, recruited using a convenience sampling technique, from February 25 to May 24, 2016 at the cardiology clinic of Ayder Comprehensive Specialized Hospital. Descriptive, inferential and Kaplan–Meier ‘tolerability’ analyses were employed. All patients were taking only enalapril as part of their angiotensin converting enzyme inhibitor treatment. According to the 2013 American College of Cardiology/American Heart Association guideline, about fourth-fifth (80.3%) of the patients were tolerating to the hypotensive effect of enalapril. The dose of enalapril was timely titrated (every 2–4 weeks) and was optimized for only 11.5 and 27.8% of the patients, respectively. Considering the tolerance, timely titration, and dose optimization, only 3.3% of the overall enalapril treatment was optimized. Multivariate regression results showed that the odds of having timely titration of enalapril for patients who were taking enalapril and calcium channel blockers were almost 20 times [adjusted odds ratio (AOR) = 21.68, 95% confidence interval (CI) 1.23–383.16, *p* < 0.036] more compared to patients who were taking enalapril and β-blockers. A Log Rank Chi Square result showed a 19.42 magnitude of better toleration of enalapril (*p* < 0.001) for patients who were taking enalapril for more than 1 year compared to less than a year.

**Conclusion:**

This study provides a platform for assessment of the treatment optimization practice of enalapril, which remains the pressing priority and found to be poor in the ambulatory setting, despite a better tolerability to the hypotensive effect of enalapril. We call for greater momentum of efforts by health care providers in optimizing the treatment practice to benchmark with other optimization practices.

## Background

Heart failure (HF) is a complex clinical syndrome that results from any structural or functional impairment of ventricular filling or ejection of blood [1]. It is one of the major and progressive causes of morbidity and mortality in most developed and some developing countries. Current therapeutic strategies have been designed to counter the progression of heart failure and to improve ‘meaningful’ survival by using medications that inhibit the remodeling process [2]. One of these strategies is to use angiotensin-converting enzyme inhibitors (ACEI) in HF patients, which is considered nowadays as one of the important and necessary steps towards an effective management of patients with HF [3].

The available evidence suggests that in chronic HF, high doses of ACEI are more effective than low ones. The current recommended clinical approach is to target ACEI dosing regimens to be similar to those used in the clinical trials, which demonstrated mortality and morbidity benefits. When titrated appropriately, ACEI are generally well tolerated and target doses can be achieved and maintained in the majority of patients with HF [4, 5].

Clinical practice guidelines, published by both the Agency for Health Care Policy and Research, and the American College of Cardiology Foundation/American Heart Association (ACCF/AHA), reflect the findings of these studies. According to these guidelines, every effort should be made to increase the dose of ACE inhibitors to the target doses shown in clinical trials to decrease mortality and morbidity with close monitoring when managing chronic HF [3, 6–8]. Moreover, studies such as the prospective evaluation by Messner Pellenc found that a daily dose of 20 mg of enalapril could be reached in a high proportion of patients with HF at good tolerability and improved outcomes [9]. The target doses used in clinical trials were 10 mg ramipril per day, 20–40 mg enalapril per day, 150 mg captopril per day, 10–35 mg lisinopril per day or 4 mg trandolapril per day [4]. Concerning the algorithm for HF management, we followed the same algorithm as outlined in ACCF/AHA and World Health Organization (WHO) guidelines [1, 10].

Regarding tolerability of ACEI, renal function, serum potassium, and signs and symptoms of a cough and angioedema should be assessed within 1–2 weeks of initiation of therapy and periodically thereafter [1, 5, 8, 11–13]. Most of HF patients (85–90%) can tolerate ACEI [1]. However, a study done in Sweden reported that 77% of the patients experienced angioedema within the first 3 weeks after starting treatment [11].

A number of prospective observational studies have reported that patients with HF discharged from hospital and maintained on ‘high’ ACEI doses had improved clinical outcomes compared to those receiving low-dose therapy. The benefits include improved patients’ symptom status, low rates of death and re-hospitalization, thus incurring lower costs [14–16]. Lack of ACEI treatment optimization significantly affects these beneficial treatment outcomes for patients with HF [17]. However, the practice of treatment optimization for this important class of medications is not assessed so far in Ayder Comprehensive Specialized Hospital (ACSH). This study was conducted in identifying the gaps in the implementation of optimal dosing of ACEI in the treatment of adult ambulatory HF patients in ACSH.

## Methods

A hospital-based cross-sectional study was employed at the cardiac unit of ACSH, Northern Ethiopia from February 25 to May 24, 2016. This institution is a public hospital that gives inpatient and outpatient services for millions of population in Tigray region and nearby regions. Heart failure patients with severe illness are usually admitted and treated as inpatients while stable patients receive care chronically as ambulatory or outpatients. The source population was all adult ambulatory HF patients who were taking HF medications. The study population in this study was all adult ambulatory HF patients who were obtaining services at the cardiology unit of ACSH and whose treatment regimen was ACEI.

Approximately 256 ambulatory HF patients who were taking ACEI regularly visit the cardiac clinic. Considering 1.96 for the standard normal variable with a 5% level of significance, 80% power of the study, 95% confidence interval (CI), 5% margin of error and 10% contingency, the sample size was calculated to be 61. A convenient sampling technique was employed to select the samples from the study population.

**Fig. 1:**
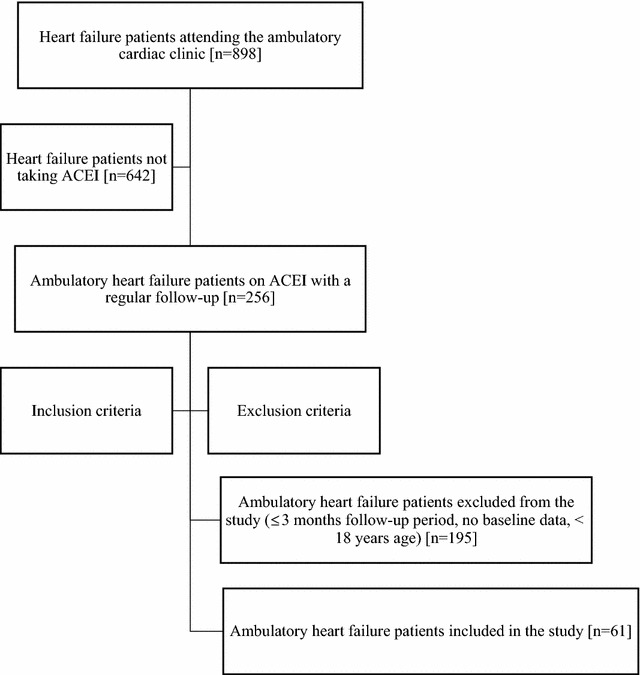
Patient flow diagram that describes the sample size calculation. ACEI, angiotensin converting enzyme inhibitors; n, number

Ambulatory HF patients who were taking ACEI as part of their treatment for at least 3 months, whose baseline information was clearly depicted in their medical records, who had a regular follow-up at the clinic and who were 18 years old and above were included in the study. On the other hand, ambulatory HF patients who were using ACEI for < 3 months, with no baseline data and younger than 18 years of old were excluded from the study. The overall patient selection process is summarized in Fig. 1.

Data were collected retrospectively by three trained data collectors (2 nurses and 1 pharmacist) through reviewing the patients’ medical records for a period of 3 months. The structured data abstraction tool was developed according to the 2013 ACCF/AHA and WHO guidelines recommendations [1, 10]. The data abstraction tool was pre-tested in 10% of the sample size (i.e., 6 medical records). Completeness of the collected data was supervised and monitored adequately by the investigators during the data collection process.

Data analysis was carried out using Statistical Package for Social Sciences (SPSS^®^ Statistics) program version 21 (SPSS; Chicago, IL, USA). Descriptive statistics such as frequency, percentage, mean, and standard deviation (SD) were employed to summarize patient, clinical, and medication-related characteristics.

Logistic regression analysis was performed to relate independent variables to treatment optimization of ACEI. From the univariate analysis, those variables with *p* < 0.2 and clinically important factors were selected for multivariate binary logistic regression analysis. The multivariate binary logistic regression analysis was also used to assess the predictability of the independent variables for treatment optimization of ACEI and to estimate the odds ratios (OR), 95% confidence intervals (CI) and *p* values. A ‘tolerability’ analysis for ACEI was carried out using the Kaplan–Meier analytic method. A Log Rank (Mantel–Cox), Breslow (Generalized Wilcoxon) and Tarone–Ware tests were employed in the overall comparisons of the ‘tolerability’ curves. The association was declared significant for the aforementioned analyses at *p* < 0.05.

### Operational definitions

ACEI was deemed to be ‘tolerated’ if the blood pressure was greater than 80/60 mmHg, serum creatinine was less than 3 mg/dL, serum potassium was < 5.5 mEq/L and no history or current complaint of a cough or angioedema. Otherwise, the ACEI was deemed to be ‘non-tolerated’. Angioedema was defined as swelling of lips, mouth, tongue, or airway in patients receiving ACEI therapy, where no other clinical cause was identified and where there were no recurrent symptoms following cessation of the drug [1, 5].

The time interval between the dose titration was said to be ‘appropriate’ if the tolerated dose was timely titrated (every 2–4-week interval). Time for a dose titration was considered as ‘inappropriate’ if the dose was not titrated within 2–4 weeks of the time interval. A dose of ACEI was said to be ‘optimized’ when once the drug is initiated and has been titrated up by 12.5 mg three times daily for captopril, 10 mg two times daily for lisinopril, and 2.5–5 mg daily for enalapril to the higher doses according to 2013 ACCF/AHA guideline. Lastly, the overall ACEI treatment was deemed to be ‘optimized’ when the dose of ACEI was optimized with timely titration, and the patient tolerated the ACEI [1].

## Results

The socio-demographics and clinical characteristics of the study participants are summarized in Table 1. According to the medical records of the study patients, more than half (57.4%) of the patients were males and were dwelling in urban areas (68.9%). Besides this, about one-third (34.4%) of the patients were in the age group of 53–69 years (mean 51.77 years; SD ± 17.56 years; range 19–83 years). Forty-five percent of the study patients had evidence of hypertension as a co-morbid disease followed by diabetes mellitus (34.4%). With reference to the cause of HF, 57.4% of the documented cause of HF were rheumatic valvular heart disease, followed by hypertension (28%). Furthermore, 42.6% of the patients had HF with reduced ejection fraction. For the majority (n = 28, 62.2%) of the study participants, the ejection fraction was 50%. The evidence for classifying the ejection fraction was based on the hospital’s reference value (60 ± 10%). In view of that, patients with ejection fraction ≤ 40% were categorized as HF with reduced ejection fraction (Table 1).

**Table 1:** Socio-demographic and clinical characteristics of heart failure patients taking ACEI at the cardiac clinic of Ayder Comprehensive Specialized Hospital, 2016

Variable	N (%)
Sex	
Male	35 (57.4)
Female	26 (42.6)
Age (years)	
19–35	13 (21.3)
36–52	15 (24.6)
53–69	21 (34.4)
≥ 70	12 (19.7)
Place of residence	
Rural	19 (31.1)
Urban	42 (68.9)
Type of comorbidity (n = 56)	
Hypertension	28 (45.0)
Diabetes mellitus	21 (34.4)
Anemia	1 (1.6)
Others^a^	6 (9.8)
Cause of heart failure	
Rheumatic valvular heart disease	35 (57.4)
Hypertension	13 (21.3)
Others^b^	13 (21.3)
Comorbidity disease	
No	30 (49.2)
Yes	28 (45.9)

^a^Myocardial infarction, coronary artery disease, ischemic heart disease, thyrocardiac disease

^b^Thyrotoxicosis, ST elevation myocardial infarction, rheumatic heart disease, HIV/AIDS

**Fig. 2:**
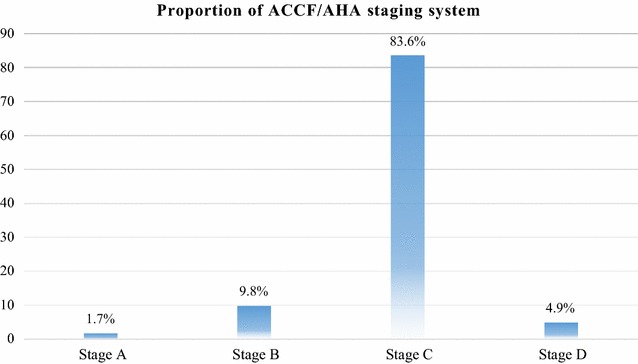
Proportion of ACCF/AHA staging system among heart failure patients taking ACEI at the cardiac clinic of Ayder Comprehensive Specialized Hospital, 2016. ACC, American College of Cardiology; AHA, American Heart Association

**Fig. 3:**
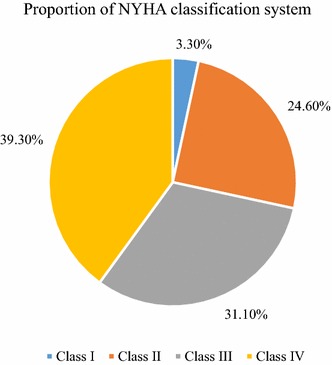
Proportion of NYHA classification system among heart failure patients taking ACEI at the cardiac clinic of Ayder Comprehensive Specialized Hospital, 2016. NYHA, New York Heart Association

**Fig. 4:**
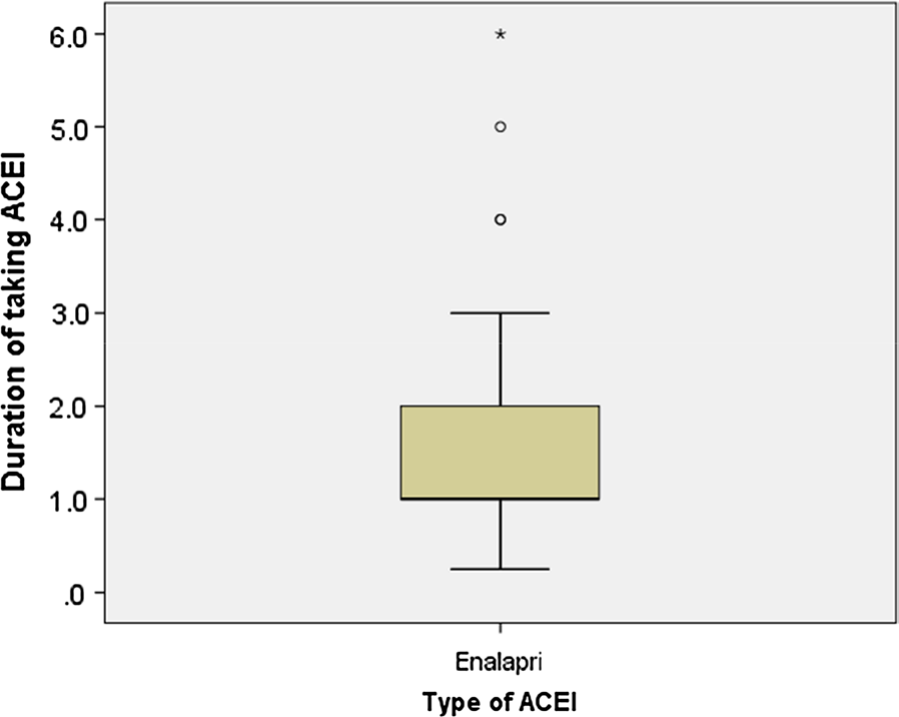
Box-and-whisker plots of type of ACEI in relation to the duration of taking ACEI among heart failure patients attending Ayder Comprehensive Specialized Hospital, 2016. ACEI, angiotensin converting enzyme inhibitors

The mean duration of diagnosis of HF for the patients was 1.8 years (SD ± 1.3 years; range 3 months to 6 years). According to the ACCF/AHA staging system, above fourth-fifth (83.6%) of the patients had stage “C” HF (Fig. 2). In addition to staging, the most prevalent category of NYHA class was class IV, accounted for 39.3%, followed by class III (31.1%) (Fig. 3). All of the study participants were using only enalapril as part of their ACEI treatment. The mean duration of taking this medication was 1.5 years (SD ± 1.2 years; range 3 months to 6 years; 95% CI 1.2–1.8) (Fig. 4).

**Fig. 5:**
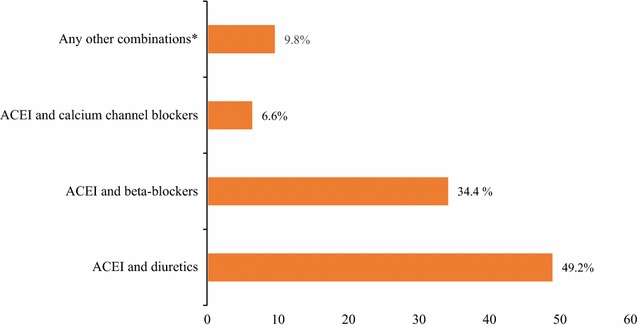
Medications profile among ambulatory heart failure patients at the cardiac clinic of Ayder Comprehensive Specialized Hospital, 2016. *ACEI and statins, ACEI and anticoagulants, ACEI and digoxin. ACEI, angiotensin converting enzyme inhibitor

All of the study patients were using one or more other types of HF medications in addition to enalapril. The combination of the ACEI and diuretics accounted for about half (49.2%) of the prescribed medications followed by a combination of the ACEI and β-blockers (34.4%) (Fig. 5).

### Treatment optimization and associated factors

**Fig. 6:**
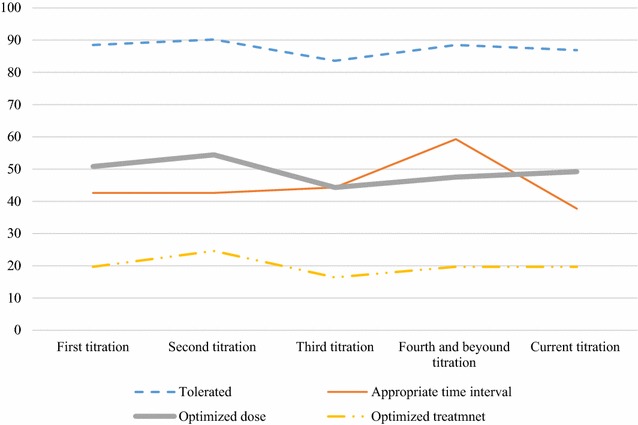
Comparison of the toleration, timely titration, dose optimization an overall ACEI treatment optimization during different titration periods among heart failure patients attending the cardiac clinic of Ayder Comprehensive Specialized Hospital, 2016. ACEI, angiotensin converting enzyme inhibitor

Regarding the toleration using blood pressure as a monitoring parameter, about fourth-fifth (80.3%) of the study subjects were tolerating to this effect during the periods of apparent titration periods but few patients were not tolerating their medication during the third titration period. On the contrary, for the majority (88.5%) of the study subjects, their dose was not timely titrated and near three-fourth (72.1%) of the patients’ dose was not optimized during these titration periods. The majority of the patients had a timely titration of ACEI during fourth and beyond titration periods. Doses of ACEI were not optimized during the third titration period, compared to the fourth and beyond titration periods. Considering the above three factors (tolerance, timely titration, and dose) in combination, the ACEI treatment was not optimized for almost all (96.7%) of the patients (Table 2 and Fig. 6).

**Table 2:** Comparison of the toleration, timely titration, dose optimization and overall ACEI treatment optimization during different periods of titration among heart failure patients taking ACEI at the cardiac clinic of Ayder Comprehensive Specialized Hospital, 2016

	First titration	Second titration	Third titration	Fourth and beyond titration	Current titration	Overall status
Was the ACEI tolerated during?						
Yes^a^, n (%)	54 (88.5)	55 (90.2)	51 (83.6)	54 (88.5)	53 (86.9)	49 (80.3)
No, n (%)	7 (11.5)	6 (9.8)	10 (16.4)	7 (11.5)	8 (13.1)	12 (19.7)
Was the ACEI timely titrated during?						
Yes, n (%)	26 (42.6)	26 (42.6)	27 (44.3)	24 (39.3)	23 (37.7)	7 (11.5)
No, n (%)	35 (57.4)	35 (57.4)	34 (55.7)	34 (55.7)	38 (62.3)	54 (88.5)
Was the dose of ACEI optimized during?						
Yes, n (%)	31 (50.8)	35 (57.4)	27 (44.3)	29 (49.2)	30 (49.2)	17 (27.9)
No, n (%)	30 (49.2)	26 (42.6)	34 (55.7)	32 (52.5)	31 (50.8)	44 (72.1)
Was the overall ACEI treatment optimized?						
Yes, n (%)	12 (19.7)	15 (24.6)	10 (16.4)	12 (19.7)	12 (19.7)	2 (3.3)
No, n (%)	49 (80.3)	46 (75.4)	51 (83.6)	49 (80.3)	49 (80.3)	59 (96.7)

ACEI, angiotensin converting enzyme inhibitor

^a^The cut off point for “yes” was 5 and for “no” was 0–4, considering the five titration periods from first up to the current

The results of univariate and multivariate logistic regression analysis showed that no factor was significantly associated with the toleration, dose optimization and overall treatment optimization of ACEI at *p* < 0.05. Moreover, the following factors that interact with heart failure were assessed for their association with tolerability of ACEI. These were: (1) cardiac events: coronary heart disease, atrial fibrillation, uncontrolled hypertension, and arrhythmia; (2) non-cardiac events: pulmonary infections, pulmonary emboli, diabetes mellitus, worsening renal function, hyperthyroidism, anemia, and pregnancy; and (3) drugs: negative inotropic medication (azithromycin, β-blockers, non-dihydropyridine calcium channel blockers, and itraconazole), direct cardiotoxics (anticancers, ethanol, and amphetamine) and drugs with sodium and water retaining properties (non-steroidal anti-inflammatory drugs, COX-2 inhibitors, glucocorticoids, and sodium-containing drugs). These factors were not found to have any statistically significant association, at the crude level, with the ACEI toleration.

On the other hand, the results of regression analysis for factors associated with timely titration are summarized in Table 3 after controlling the independent variables and incorporating those variables with *p* value less than 0.2 (including duration of diagnosis and types of other concomitant medications) into the multivariate logistic regression analysis. From the multivariate analysis, the odds of having timely titration of ACEI for patients who were taking ACEI and calcium channel blockers were almost twenty times (AOR = 21.68, 95% CI 1.23–383.16, *p* = 0.036) more compared to patients who were taking ACEI and β-blockers (Table 3).

**Table 3:** Results of multivariate logistic regression analysis for factors associated with timely titration of ACEI among heart failure patients at the cardiac clinic of Ayder Comprehensive Specialized Hospital, 2016

Variable	Timely titration		COR, 95% CI	*p* value	AOR, 95% CI	*p* value
	No, n (%)	Yes, n (%)				
Duration of heart failure diagnosis (year)						
≤ 1	25 (80.6)	6 (19.4)	1.00		1.00	
> 1	29 (96.7)	1 (3.3)	0.144 (0.02–1.78)*	0.082	0.102 (0.01–1.22)*	0.071
Type(s) of combination medications						
ACEI and beta-blockers	28 (93.3)	2 (6.7)	1.00		1.00	
ACEI and CCB	2 (50.0)	2 (50.0)	14.00 (1.23–158.84)**	0.033	21.68 (1.23–383.16)**	0.036
ACEI and diuretics	19 (90.5)	2 (9.5)	1.47 (0.19–11.39)	0.710	1.25 (0.15–10.22)	0.835
Any other combinations^a^	54 (88.5)	7 (11.5)	2.80 (0.212–37.03)	0.434	2.86 (0.19–43.34)	0.447

AOR, Adjusted odds ratio; CCB, calcium channel blockers; CI, Confidence interval; COR, Crude odds ratio

* Statistically significant at *p* < 0.1

** Statistically significant at *p* < 0.05

^a^Any other combinations: ACEI and statins, ACEI and anticoagulants, ACEI and digoxin

### Kaplan–Meier ‘tolerability’ analyses

**Fig. 7:**
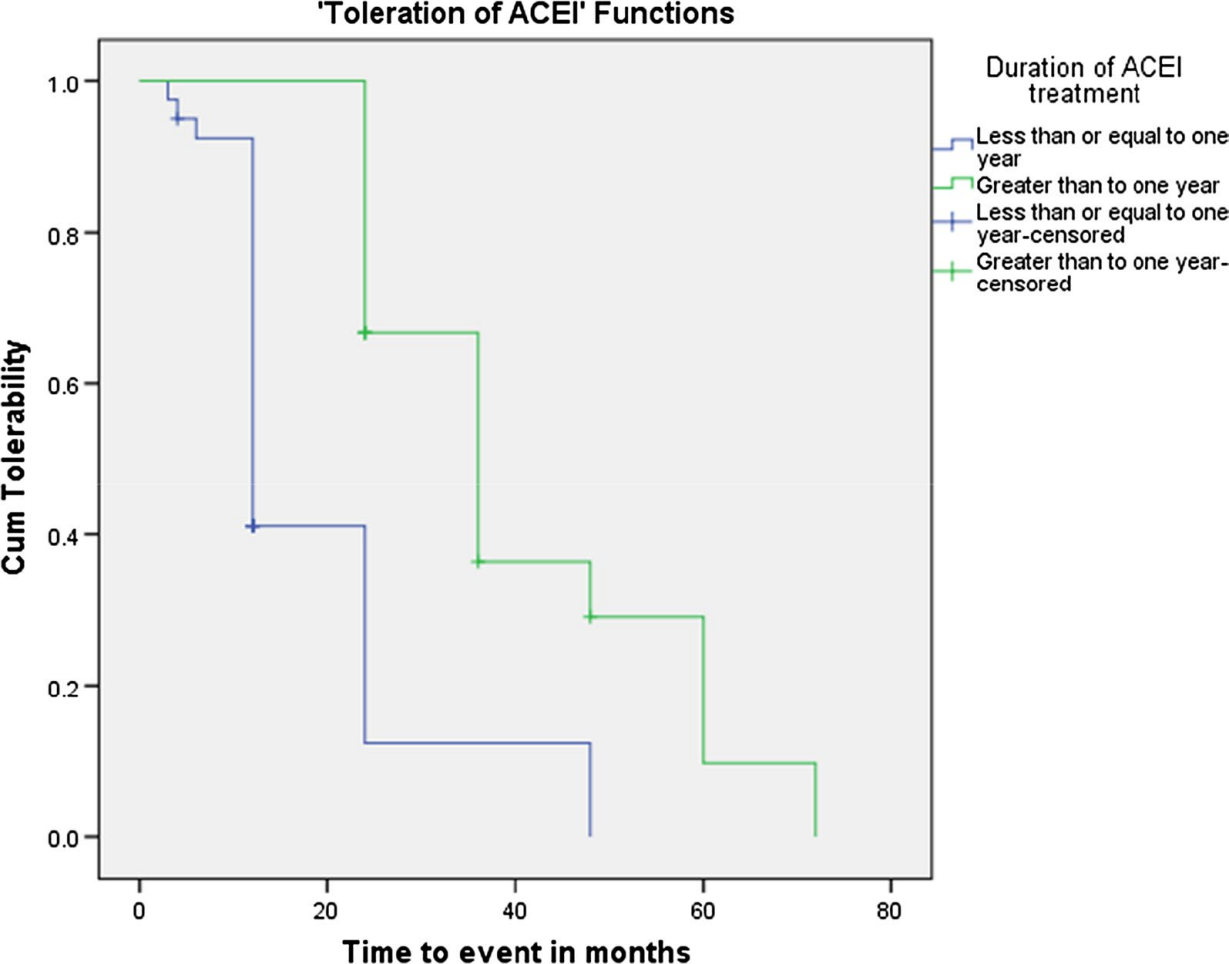
Kaplan–Meier curve for toleration of ACEI among heart failure patients by duration of ACEI treatment at the cardiac clinic of Ayder Comprehensive Specialized Hospital, 2016. ACEI, angiotensin converting enzyme inhibitor

The event of interest during the Kaplan–Meier (KM) ‘tolerability’ analysis method in this study was ACEI treatment toleration and the units of measurement along the x-axis were the duration of taking ACEI in months. Patients who were taking ACEI for more than 1 year were tolerating their medication in a better way than patients who were taking ACEI for less than a year. The test of equality of survival distributions for the duration of ACEI treatment showed the Chi Square results of 19.42, 21.78 and 21.47 for the Log Rank (Mantel–Cox) (*p* < 0.001), Breslow (Generalized Wilcoxon) (*p* < 0.001) and Tarone–Ware (*p* < 0.001) respectively (Fig. 7).

**Fig. 8:**
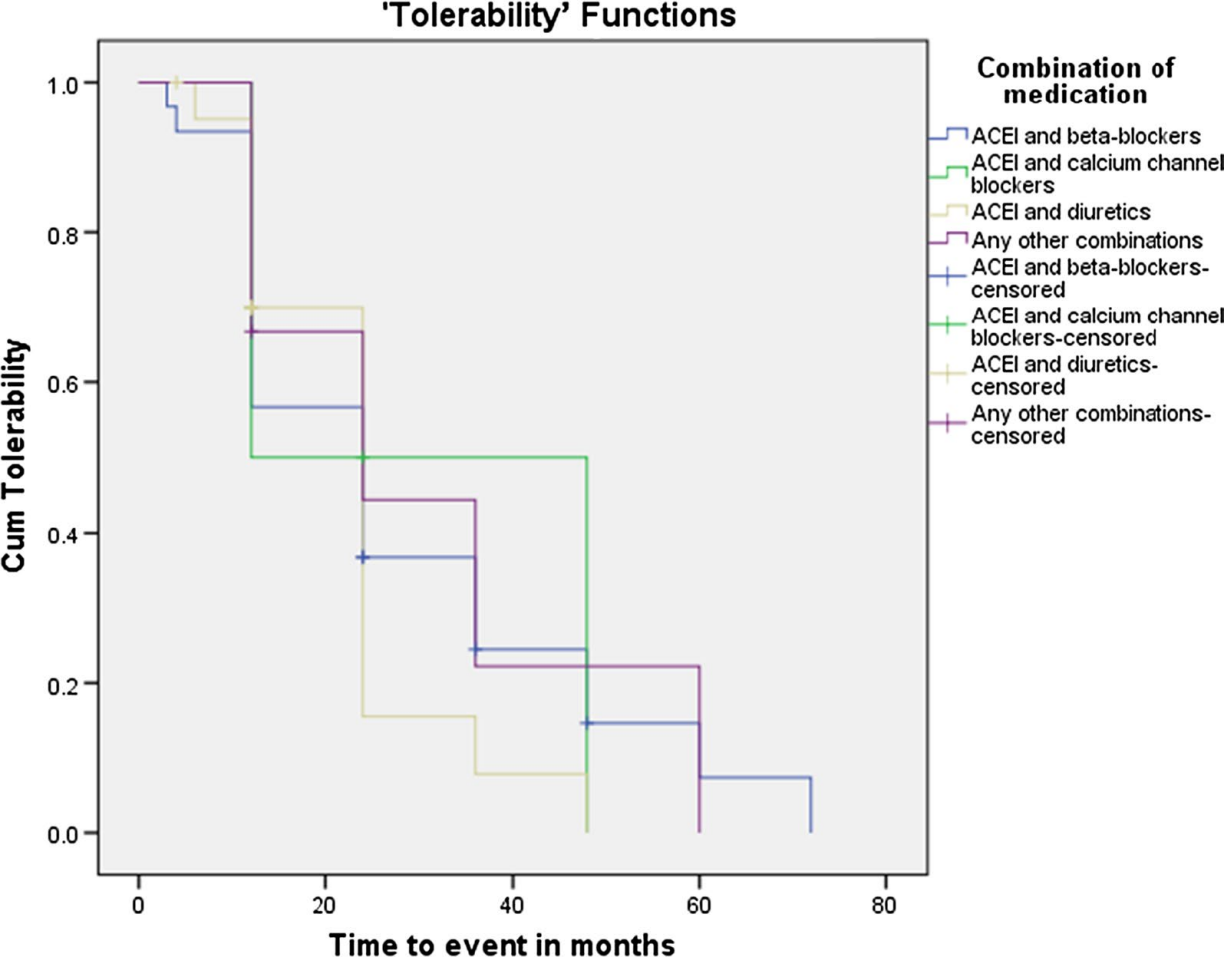
Kaplan–Meier survival curve for toleration of ACEI among heart failure patients by types of medications at the cardiac clinic of Ayder Comprehensive Specialized Hospital, 2016. Any other combinations: ACEI and statins, ACEI and anticoagulants, ACEI and digoxin. ACEI, angiotensin converting enzyme inhibitor

A comparison of KM survival curves for the types of HF medications revealed that none of the combinations of HF medications provide any effect on the toleration of the medications by the study participants. The test of equality of survival distributions for the different types of heart failure medications showed the Chi Square results of 1.204, 0.878 and 0.933 for the Log Rank (Mantel–Cox) (*p* = 0.752), Breslow (Generalized Wilcoxon) (*p* = 0.831) and Tarone–Ware (*p* = 0.817) respectively (Fig. 8).

## Discussions

The study assessed treatment optimization practice of ACEI among ambulatory HF patients at the cardiac clinic of ACSH. According to 2013 ACCF/AHA recommendation, the majority (80.3%) of the study patients were tolerating the ACEI provided blood pressure as a monitoring parameter. The probable explanation for this finding might be supported by the evidence that most of the patients were maintained on low doses for a long period of time. Moreover, there might be poor patients’ awareness on reporting the potential side effects associated with ACEI. This finding (80.3% tolerance to ACEI) was congruent with studies done in the USA that reported tolerance rate of 80% [18] and Australia that reported 75% rate [19]. This similarity could be related to the maintenance of patients on a similar dose without up-titration for a long duration of the period. Another possible explanation could also be the similarity of black patients and the matched white patients in socio-demographic and clinical characteristics [20].

Concerning timely titration of ACEI, the majority of patients’ medication was not timely titrated (that is, every 2–4 weeks) as per the recommendations. The absence of concordance on the selection of appropriate appointment date between the patients and physicians could be the possible explanation for this finding. Likewise, the medications for patients with longer duration of diagnosis was not timely titrated as substantiated by the finding that patients whose HF diagnosed for the duration of fewer than 1.6 years had 19.4% of timely titration compared to 0% timely titration for patients with 3.26 and above years’ duration. This might be associated with the resistance of physicians on consideration of the essentiality of upward titration of ACEI and might be due to the high ratio of patients per healthcare professional which could hinder the quality of medical as well as pharmaceutical care secondary to the absence of clinical pharmacy specialist at the cardiology clinic.

Besides the above findings, the doses of ACEI were not optimized for the majority of the study participants (72.9%). The patients in this study were taking a low dose of ACEI for longer periods of time without proper titration. This poor dose optimization practice corresponded with a study done in England that reported 75% [21]. The probable reason for this finding might be attributed to the poor quality of health care due to less team-work and lower involvement of clinical pharmacists in the hospital setting and the absence of updated hospital guidelines.

Moreover, the reason why patients’ doses were not optimized were allied to fear of adverse effects and the physicians claimed that patients becoming intolerant to higher doses. Despite the differences in the definition of ‘optimal’ doses of ACEI in different clinical settings, a number of surveys suggest that clinicians often prefer the use of low doses of ACEI, and the perception that low doses are as effective as high ones are quite prevalent. In addition, clinicians rarely titrate ACEI dose according to blood pressure [4].

The overall treatment in the present finding was not optimized because the dose was not optimized and timely titrated in most of the patients, and some of the patients were not tolerating the medications. This leads to the overall poor treatment optimization practice in the hospital despite the obvious favorable effects of ACEI therapy using a cascaded higher dose. Tailoring therapy to achieve a desired neurohormonal response and improve therapeutic outcomes have been demonstrated [22]. Unless optimally titrated, exacerbation of heart failure occurs commonly in these patients and contribute to the compromised quality of life for these patients [23]. There are multitudes of reasons given for why patients were either not on ACEI or at low doses of them objecting the optimum titration. In summary, the potential obstacles may be attributable to fear of adverse effects [24], patients’ inconvenience to appointment dates selected; high patient load with small number of physicians; absence of physicians’ team-work or less team-work spirit with clinical pharmacists and other health care providers; less access and awareness to the up-to-date guidelines by the clinicians; and physicians’ low knowledge, attitude and practice towards ACEI treatment optimization.

The multivariate binary logistic regression analysis showed that the combination of medications was only found to be significantly associated with timely titration. Accordingly, patients who were taking ACEI and calcium channel blockers were more likely to have timely titration compared to patients who were taking ACEI and β-blockers. This might be related to side effects of calcium channel blockers, which are common and easily identifiable by the patients. Therefore, the patients preferred to report these side effects and likely to have a short duration of appointment than patients who were taking β-blockers. On the other hand, most of the socio-demographics and clinical characteristics of the study participants were not found to be significantly associated with timely titration, dose optimization, and overall treatment optimization.

The Kaplan–Meier (KM) ‘tolerability’ analysis method showed that patients who were taking ACEI for more than 1 year were tolerating their medication in a better way than patients who were taking ACEI for less than a year. The majority of the side effects of ACEI is seen in the early phases of treatment and then wanes with the progression of time.

There was a certain limitation in this study. The cross-sectional nature of this study did not allow follow-up of the study participants, which could have provided a better design for identifying the factors associated with the treatment optimization.

## Conclusion

This study provides a platform for assessment of the treatment optimization practice of ACEI (enalapril), which remains the pressing priority and found to be poor in the ambulatory setting, despite a better tolerability to the hypotensive effect of enalapril. The combination of enalapril and calcium channel blockers is found to contribute positively to timely titration. Accordingly, we call for greater momentum of efforts by health care providers in optimizing the treatment practice to benchmark with other optimization practices in order to improve the heart failure management.

## Abbreviations

ACCF/AHA: American College of Cardiology Foundation/American Heart Association
ACEI: angiotensin converting enzyme inhibitors
ACSH: Ayder Comprehensive Specialized Hospital
AOR: adjusted odds ratio
CCB: calcium channel blockers
CI: confidence interval
COR: crude odds ratio
HF: heart failure
NYHA: New York Heart Association
WHO: World Health Organization
